# The Two Main Forms of Histiocytic Sarcoma in the Predisposed Flatcoated Retriever Dog Display Variation in Gene Expression

**DOI:** 10.1371/journal.pone.0098258

**Published:** 2014-06-02

**Authors:** Kim M. Boerkamp, Frank G. van Steenbeek, Louis C. Penning, Marian J. A. Groot Koerkamp, Dik van Leenen, Manon Vos-Loohuis, Guy C. M. Grinwis, Gerard R. Rutteman

**Affiliations:** 1 Faculty of Veterinary Medicine, Department of Clinical Sciences Companion Animals, Utrecht University, Utrecht, The Netherlands; 2 Molecular Cancer Research, University Medical Centre Utrecht, Utrecht, The Netherlands; 3 Faculty of Veterinary Medicine, Department of Pathobiology, Utrecht University, Utrecht, The Netherlands; Colorado State University, United States of America

## Abstract

Examination of gene functions in specific tumor types improves insight in tumorigenesis and helps design better treatments. Due to the rarity of histiocytic/dendritic cell sarcoma in humans, it is difficult to accrue such knowledge. Therefore, comparative research of these cancers in predisposed dog breeds, such as the Flatcoated retriever, can be of value. Histiocytic sarcoma in the dog can be grouped into a soft tissue- and visceral form. The soft tissue form at first is localized, while the visceral form progresses more quickly to a terminal state, which might be related to variations in gene expression. Microarray analyses were performed on fresh-frozen tissue from Flatcoated retrievers with either soft tissue- or visceral histiocytic sarcoma. Expression differences of ten most significantly differentially expressed genes were validated with quantitative real-time PCR (q PCR) analyses. Q PCR analyses confirmed the significantly aberrant expression of three of the selected genes: *C6* was up-regulated; *CLEC12A* and *CCL5* were down-regulated in the visceral histiocytic sarcoma compared to the soft tissue form. The findings of our study indicate that these two forms of histiocytic sarcoma in the dog display a variation in gene expression and warrant analysis of functional changes in the expression of those genes in these rare sarcomas in man.

## Introduction

Histiocytic malignancies include dendritic cell- and histiocytic sarcomas and disseminated Langerhans cell histiocytosis [1]. In humans, the frequency of this group of diseases is very low [2]. This hampers an evidence based therapeutic approach for instance based on selective inhibition of specific signal transduction pathways. Canine histiocytic sarcoma (HS) resemble the human histiocytic malignancies [3]–[6]. As for such human cancers, canine HS involves the proliferation of members of both histiocytic lineages; dendritic cells and macrophages [5], [6]. The dog is genetically closer related to man than mice [7], [8] and the study of genetic changes of spontaneous cancers in the dog therefore has high comparative value [3], [9], in particular for cancers that are rare in the human such as histiocytic malignancies [10], [11]. Research could lead to more insight in the pathogenesis of this disease and could facilitate the identification of therapeutic targets valuable for both species [4], [7], [12]–[15].

In the dog, the limited genetic flow within breeds is responsible for specific breed traits including disease predispositions. However, the disease predispositions resulting from selective inbreeding can be studied for the benefit of affected breeds as well as for humans in which the rarity of diseases such as histiocytic malignancies hampers scientific progress. The Flatcoated retriever (FCR) has a strongly increased risk for HS development [16], [17]. In dogs [3], [4], [16] and humans [10], [11], histiocytic malignancies are grave conditions though the prognosis varies between subtypes [1]. HS has two common subtypes. Localized, soft tissue histiocytic sarcoma (STHS), manifests itself as a tumor arising in the deeply seated soft tissues of limbs, often in association with joints. In this form, chemotherapy as an adjunct to tumor resection can improve survival in some dogs that suffer from STHS [18]. The prognosis for the second form; visceral histiocytic sarcoma (VHS), which is a multifocal and disseminated form that is manifested in internal organs, uniformly is very poor [3], [16], [17], [19], [20]. There is presently no immunohistochemical method available to definitively differentiate between STHS and VHS and some have stated that VHS and STHS represent two different stages along a continuum of the same disease [5], [21].

Our previous research of HS in Flatcoated retrievers has provided evidence that the expression of nine common genes is altered when comparing both STHS and VHS with normal spleen; indicating a common ground for the general development of HS [15]. As a next step we compared the two forms of HS with one another. In this additional study we provide evidence that, in addition to common changes in STHS and VHS compared to healthy tissues described previously, there are some marked genetic differences between these two common forms of histiocytic malignancies.

## Materials and Methods

The experimental protocol (ID 2007.III.08.110) was peer-reviewed by the scientific committee of the Department of Animals in Science & Society, Utrecht University, The Netherlands, and approved by the Animal Experiments Committee of the Academic Biomedical Centre, Utrecht, The Netherlands. The Animal Experiments Committee based its decision on ‘De Wet op de Dierproeven’ (The Dutch ‘Experiments on Animals Act’, 1996) and on the ‘Dierproevenbesluit’ (the Dutch ‘animal experiments decree’, 1996). Both documents are available online at http://wetten.overheid.nl.

### Case recruitment and histopathological evaluation

As mentioned in the earlier study [15], all tumor samples were confirmed as being spontaneously occurring histiocytic malignancies obtained from (previously untreated) Dutch family-owned FCR. All samples were obtained with informed owner consent. Case selection and obtainment of tissues were similar as mentioned in the previous study. As a common reference pool a multitude of canine organs (testis, liver, spleen, prostate, duodenum, lung, kidney and brain) from healthy crossbreeds (n = 8) was used [15].

Patient details are listed in Table 1.

**Table 1:** Patient details.

Name	Sex	Pathology	AO (yrs)	Site(s)	Microarray/PCR
D1, TJ	MN	STHS	7.7	shoulder	Y/Y
D3, UH	FN	STHS	8	shoulder	Y/Y
D4, BS	MN	STHS	6.5	elbow	Y/Y
D5, BaS	M	STHS	7.6	knee	Y/Y
D6, TV	MN	STHS	8.1	elbow	Y/Y
D7, DV	MN	STHS	11	shoulder	Y/Y
DX, YM	MN	STHS	9.7	shoulder	N/Y
D2, BE	M	VHS	9.4	liver/spleen/**lnn abd**	Y/Y
D8, DW	M	VHS	9.5	spleen/**lnn abd**	Y/N
D9, JV	FN	VHS	8.9	lung/**lnn mediast**	Y/Y
D11, BT	F	VHS	8.5	**lung**	Y/Y
D12, AG	M	VHS	7.3	**lung**/spleen/kidney	Y/Y
D13, TR	FN	VHS	7.9	lung	Y/Y
D14, SG	F	VHS	4.1	lung/**lnn mediast**	Y/Y
DX, SC	MG	VHS	10	liver/**spleen**	N/Y

AO: Age of onset, M: male, MN: male neutered, F: female, FN: female neutered, STHS: soft tissue (localized) histiocytic sarcoma, VHS: visceral (disseminated) histiocytic sarcoma, lnn abd: abdominal lymphnodes, lnn mediast: mediastinal lymph nodes. Note: For cases with VHS the site sampled for gene expression is indicated in bold letters N = no Y = yes

### RNA isolation

RNA isolation was performed as described previously [15]. In short, approximately 30 mg of frozen tumor was disrupted/lysed and homogenized and total RNA was isolated and treated with DNase using the RNeasy mini kit (Qiagen, The Netherlands) according to the manufacturer's protocol. Quantity and integrity were assessed with the Bioanalyzer Agilent BioAnalyzer-2100 (Bioanalyzer, Agilent Technologies, Santa Clara, CA) in combination with an RNA 6000 Pico-LabChip. The average RNA integrity number 8.5 (range: 7.2–9.8) was found to be appropriate [22]. RNA concentration was quantified using a NanoDrop ND-1000 (Isogen Life Science) spectrophotometer.

### Expression profiling

Expression profiling was performed as described previously [15]. In short, RNA was labeled twice and hybridized against the common reference RNA on dual channel arrays, with RNA amplifications and labeling being performed on an automated system (Caliper Life Sciences NV/SA, Belgium) as described previously [23]. Dye swap of Cy3 and Cy5 was performed to reduce dye bias. Hybridizations were done on a HS4800PRO system supplemented with QuadChambers (Tecan Benelux B.V.B.A.) using 500–1000 ng labeled cRNA per channel as described [24]. After automated data extraction using Imagene 8.0 (BioDiscovery), printtip Loess normalization was performed [25] on mean spot-intensities. Dye-bias was corrected based on a within-set estimate as described [26]. Data were analyzed using ANOVA [27] (R version 2.2.1/MAANOVA version 0.98–7) (http://www.r-project.org/). Briefly, both tumourgroups were compared through the common reference channel. *P*-values were determined by a permutation F2-test, in which residuals were shuffled 5000 times globally. Thus analyzed 191 gene probes with *P*<0.05 after family wise error correction (FWER) were considered significantly changed.

### Functional annotation

To examine whether certain pathways are over- or under-represented in the gene list, all genes differentially expressed between STHS and VHS, were introduced into DAVID (http://david.abcc.ncifcrf.gov/).

### Quantitative real time PCR; gene selection

Following the outcome of the microarray expression profiling, 11 genes were selected; namely C-type lectin domain family 12, member A (*CLEC12A*); C-C motif chemokine 5 Precursor (Small-inducible cytokine A5) (*CCL5_CANFA*); Asporin (*ASPN*); CD9 molecule (*CD9*); Transketolase-like 1, transcript variant 1 (*TKTL1*); Complement component 6, transcript variant 1(*C6*); S100 calcium binding protein A12 (*S100A12*); Immunoglobulin J polypeptide (*IGJ*); S100 calcium binding protein A8 (*S100A8*); Phytanoyl-CoA dioxygenase, peroxisomal like (*PHYH*). Selection of these genes was based on the ones most significantly differently expressed on the micro-array chip and their fold changes.

Details of the qPCR reactions and primer sequences are depicted in Table 2. Delta Ct method, using efficiencies between 95.0 and 104.8%, was used for both the reference- as well as the target genes.

**Table 2:** QPCR primers for genes of interest (based on Microarray analyses) and reference genes (efficiencies varied between 95.0 and 104.8%).

Gene name	ENSID	Primer sequence	Forward/reverse	Amplicon size (bp)	AnnealingT (°C)
*CLEC12A*	ENSCAFG00000025113	AAATGCCAGCCTGTTGAC	F	110	61
		TGGTAATCTCTGTCATACTTGGG	R		
*CCL5_CANFA*	ENSCAFG00000018171	TATGCCTCAGACACCACAC	F	119	63
		GACAAAGACGACTGCTGG	R		
*ASPN*	ENSCAFG00000002307	CCACGAGTCAGAGAAATACAC	F	135	59
		GGCAGAAGTCATTCACTCC	R		
*CD9*	ENSCAFG00000015172	TGTGCTGTCATCCATCAC	F	118	57
		TGCCAAATATCATCACTACGG	R		
*TKTL1*	ENSCAFG00000019451	ATGAGATACAAACAGGAGGAC	F	136	61
		CCAGTATATGCCATCCCAC	R		
*C6*	ENSCAFG00000018598	CCGTTGTGATTGACTTTGAG	F	88	61
		CTTTCTGAGGTTGTTCCGT	R		
*S100A12*	ENSCAFG00000023324	AAAGGGTGAGATGAAGCAG	F	156	61
		CACAACAGAAACCAGGGA	R		
*IGJ*	ENSCAFG00000002911	CCTTCTCCCGATGATCCT	F	120	63
		GGTACACAAATTTCGTTCTCAC	R		
*S100A8*	ENSCAFG00000017557	GTTTACCACAAGTACTCCCTG	F	148	63
		CCATCGCTATTGACATCCA	R		
*PHYH*	ENSCAFG00000023349	CTGAAGCCACACGATTATCC	F	112	58
		TCTCCTTTCTCCATCACGA	R		
*HPRT*	ENSCAFG00000018870	AGCTTGCTGGTGAAAAGGAC	F	104	56
		TTATAGTCAAGGGCATATCC	R		
*RPS19*	ENSCAFG00000028485	CCTTCCTCAAAAAGTCTGGG	F	95	61
		GTTCTCATCGTAGGGAGCAAG	R		
*RPL8*	ENSCAFG00000001677	CCATGAATCCTGTGGAGC	F	64	55
		GTAGAGGGTTTGCCGATG	R		
*SRPR*	ENSCAFG00000010474	GCTTCAGGATCTGGACTGC	F	81	61
		GTTCCCTTGGTAGCACTGG	R		
*RPL13*	ENSCAFG00000019840	GCCGGAAGGTTGTAGTCGT	F	87	61
		GGAGGAAGGCCAGGTAATTC	R		
*GUSB*	ENSCAFG00000010193	AGACGCTTCCAAGTACCCC	F	103	62
		AGGTGTGGTGTAGAGGAGCAC	R		
*GAPDH*	ENSCAFG00000024323	TGTCCCCACCCCCAATGTATC	F	100	58
		CTCCGATGCCTGCTTCACTACCTT	R		
*B2MG*	ENSCAFG00000013633	TCCTCATCCTCCTCGCT	F	85	61
		TTCTCTGCTGGGTGTCG	R		
*RPS5*	ENSCAFG00000002366	TCACTGGTGAGAACCCCCT	F	141	62.5
		CCTGATTCACACGGCGTAG	R		

Genes identified using microarray as being significantly different comparing Soft Tissue Histiocytic Sarcoma (STHS) and Visceral Histiocytic Sarcoma (VHS): *C-type lectin domain family 12, member A (CLEC12A); C-C motif chemokine 5 Precursor (Small-inducible cytokine A5) (CCL5_CANFA); Asporin (ASPN); CD9 molecule (CD9);Transketolase-like 1, transcript variant 1 (TKTL1); Complement component 6, transcript variant 1(C6); S100 calcium binding protein A12 (S100A12); Immunoglobulin J polypeptide (IGJ); S100 calcium binding protein A8 (S100A8); Phytanoyl-CoA dioxygenase, peroxisomal like (PHYH)* Reference genes primers for q PCR: *Hypoxanthine phosphoribosyltransferase (HPRT), Ribosomal protein S19 (RPS19) ribosomalprotein L8 (RPL8), Signal recognition particle receptor (SRPR), Ribosomal protein L13, (RPL13), glucuronidase, beta (GUSB), Glyceraldehyde-3-Phosphate Dehydrogenase (GAPDH), Beta-2-Microglobulin (B2MG), 40S ribosomal protein S5 (RPS5), Glyceraldehyde-3-phosphate dehydrogenase (GAPDH), β-2-microglobulin (B2MG), Ribosomal protein S5 (RPS5).*

### RNA isolation and cDNA synthesis

Besides tissues from all but one patient (of which the insufficient tissue remained for the validating qPCR experiment) used in the microarray experiment (six soft tissue HS and seven visceral HS), two additional samples (one soft tissue HS and one visceral HS that met the inclusion criteria) were added. Total RNA from these samples was isolated. After isolation, total RNA was treated with DNase using the RNeasy mini kit (Qiagen, The Netherlands) according to the manufacturer's protocol.

Reverse transcription (RT) was performed of all 20 samples in a 80 µl reaction using 2000 ng total RNA, 16 µl iScript Reaction mix and 4 µl iScript Reverse Transcriptase (iScript cDNA Synthesis kit, Bio Rad, Veenendaal, The Netherlands). The mixture, contain both random hexamers and oligo-dT primers,was incubated 5 min. at 25°C, 30 min. at 42°C and followed by 5 min. at 85°C. Minus RT controls were prepared from 500 ng of the same RNA under the same conditions, but without addition of reverse transcriptase.

### Reference genes and primer development

Nine reference genes were used as the non-regulated reference genes for normalization, based on their stable expression in canine tissue [28], [29] namely *ribosomal protein S19 (RPS19) hypoxanthine phosphoribosyltransferase (HPRT), ribosomal protein L8 (RPL8), signal recognition particle receptor (SRPR), and ribosomal protein L1 (RPL13), glucuronidase, beta (GUSB), Glyceraldehyde-3-Phosphate Dehydrogenase (GAPDH), Beta-2-Microglobulin (B2MG), 40S ribosomal protein S5 (RPS5).* Primers for reference genes, including their optimum annealing temperature are listed in Table 2.

Using Ensembl (Ensembl 70; CanFam3.1), through annotated transcripts, PCR primers were designed using the Perl Primer software (version 2.0.0.7) according to the parameters outlined in the Bio-Rad i-cycler manual. The specificity of each primer pair was confirmed by sequencing its product and also in qPCR by checking the meltcurve and reaction efficiency. GeNorm [30] was used to establish expression stability. Amplicon sequence-reactions were performed using BigDye v3.1 according to the manufacturer's (Life Technologies, Bleiswijk, The Netherlands) instructions on an ABI3130XL and analyzed in Lasergene (version 9.1 DNASTAR) and confirmed the specificity of each amplicon.

### Quantitative PCR

Published guidelines for the qPCR experiment were followed according to the MIQE guidelines [31]–[33]. For qPCR, the CFX detection system (Bio-Rad.) with SYBR green fluorophore was used. Reactions were performed in a total volume of 10 µl containing 5 µl of 2× SYBR green super mixes (Bio-Rad Laboratories Ltd.), 0.5 µl of each primer at 400 nM concentration, 0.8 µl of cDNA and 3.2 µl RNase and DNase free water as previously described [15], [34], [35]. Expression analysis was performed on sample duplicates in duplicate. A minus RT sample and a no template control were performed as control. Expression levels were based on Ct values normalized using the mean of seven of the nine reference genes. GAPDH and B2MG did not behave fully stable according to GeNorm [28].

A Wilcoxon rank sum test was performed to determine the significance of differential gene expression. All results were Bonferroni corrected.

## Results and Discussion

The Microarray enabled analysis of the expression of 42,034 features; however, since only 21,682 (51%) were annotated (CanFam 2.0), it is possible that important genes are missed. Gene expression profiles of the two HS forms were compared with each other to identify genes that are specific for each manifestation.

**Figure 1:**
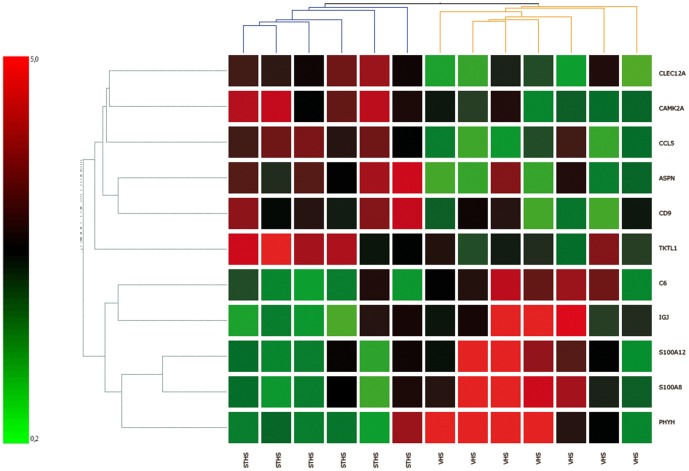
Microarray- based heatmap of 11 genes. *C-type lectin domain family 12, member A (CLEC12A); C-C motif chemokine 5 Precursor (Small-inducible cytokine A5) (CCL5_CANFA); Asporin (ASPN); CD9 molecule (CD9);Transketolase-like 1, transcript variant 1 (TKTL1); Complement component 6, transcript variant 1(C6); S100 calcium binding protein A12 (S100A12); Immunoglobulin J polypeptide (IGJ); S100 calcium binding protein A8 (S100A8); Phytanoyl-CoA dioxygenase, peroxisomal like (PHYH).*

When comparing VHS and STHS, 191 probes were significantly differentially expressed, listed in Additional Table S1. From the selection of most significant (*P*<0.003), unique probes with a threshold of log2 fold change of 1.5 (n = 19), eight were excluded for either comprising chromosomal regions (n = 3) or as clones that did not align (n = 5). The eleven genes that remained, are visualized in a heatmap (Figure 1). Their potential involvement in tumor development or behavior was considered by a literature research. All microarray gene expression data were deposited in the public data repository GEO (accession number GSE45832).

**Figure 2:**
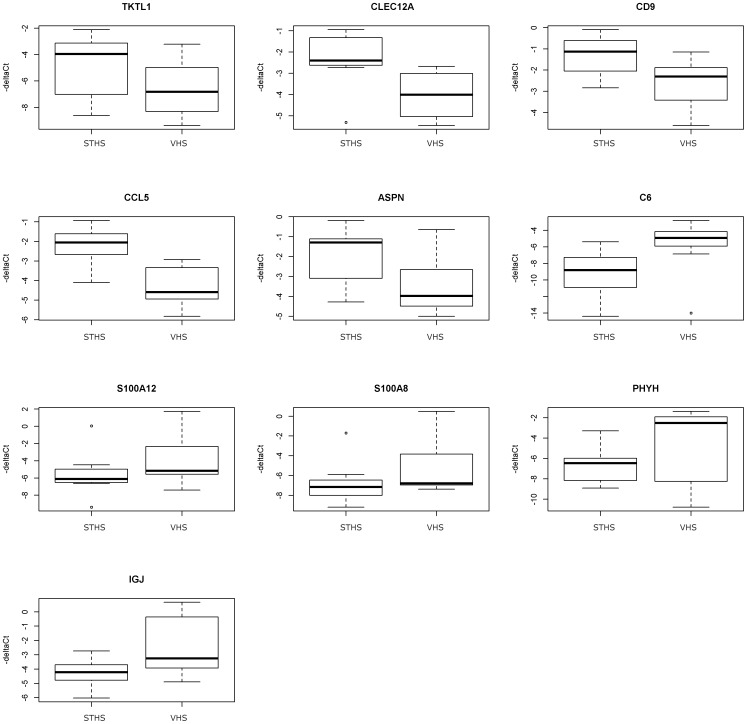
Quantitative PCR results. The upregulation or downregulation of selected genes in STHS (Soft Tissue Histiocytic Sarcoma) and VHS (Visceral Histiocytic Sarcoma). The thick black line represents the median (50th percentile) and also the first and third quartile (25th and 75th percentile respectively) are displayed.Three genes are significantly differentially expressed: *C6* was up-regulated when comparing the more aggressive visceral histiocytic sarcoma to the soft tissue form, and *CLEC12A* and *CCL5* were down-regulated when comparing the more aggressive visceral histiocytic sarcoma to the soft tissue histiocytic sarcoma. Abbreviations*: C-type lectin domain family 12, member A (CLEC12A); C-C motif chemokine 5 Precursor (Small-inducible cytokine A5) (CCL5_CANFA); Asporin (ASPN); CD9 molecule (CD9);Transketolase-like 1, transcript variant 1 (TKTL1); Complement component 6, transcript variant 1(C6); S100 calcium binding protein A12 (S100A12); Immunoglobulin J polypeptide (IGJ); S100 calcium binding protein A8 (S100A8); Phytanoyl-CoA dioxygenase, peroxisomal like (PHYH).*

QPCR confirmed and quantified the differential expression of the 11 genes selected from the micro-array data. As a result, *C6*, *S100A12*, *S100A8*, *PHYH* and *IGJ* were up-regulated and *TKTL1*, *CLEC12A*, *CD9*, *CCL5* and *ASPN* were down-regulated in VHS compared to STHS. Only for three gene products was the difference in expression significant: *C6* (*P* = 0.038), *CLEC12A* (*P* = 0.026) and *CCL5* (*P* = 0.0069) (Figure 2).

For technical reasons, no qPCR data could be obtained for *CAMK2A.*

Our observations of *CLEC12A* and *CCL5* and those made in several human cancer types, make it conceivable that these gene products play a role in HS. In humans, C chemokine ligand 5 (*CCL5*) functions as one of the natural ligands for the CC Chemokine Receptor 5 (*CCR5*). It mediates chemotactic activity in immune cells including monocytes and dendritic cells [36] *CCL5* [37], [38] and *CCR5* [39] promote breast cancer invasiveness and metastatic potential, while *CCR5* inhibition abrogates this [39]. For inflammatory breast cancer, *CCL5* is considered to constitute a prominent part of a poor prognosis signature [40]. Also in human colorectal carcinoma *CCL5* appears to stimulate cancer progression [41]. Furthermore, the *CCL5/CCR5* axis has been shown to promote cell motility in human osteosarcoma [42]. Thus, for these human cancers *CCL5* appears to be associated with a metastatic phenotype. Yet, in our study, *CCL5* was expressed at lower level in VHS than in STHS. Considering the more rapid metastatic nature of VHS, this is surprising, and this finding needs confirmation (also at protein level) in future studies. Still, a decrease in *CCL5* expression has been described in other human malignancies such as colon carcinoma when compared to normal tissue [43]. An alternative explanation for a reduction in CCL5 expression in visceral as compared to localized HS could be that a reduction in expression of *CCL5* could protect against immunosurveillance [44] and hence be related to more aggressive behavior of HS.

*CLEC12A* expression was found to be significantly lowered in VHS compared to STHS. *CLEC12A* (or *MICL*) is considered a negative regulator of granulocyte and monocyte function [45]. Activation of myeloid cells and recruitment to sites of inflammation – but not increase or decrease in the level of differentiation – was accompanied by reduced expression [46]. Whereas normal lymphocytes have no or low *MICL* expression, a relatively high expression of this gene in acute lymphoblastic leukemia was found to be associated with prolonged relapse-free survival [47]. How the reduced expression in VHS as compared to STHS relates to these findings remains to be determined, but a resulting increased migratory capacity as present in VHS could be an explanation.

*Complement component 6* (*C6*) gene expression was significantly increased in VHS as compared to STHS. In acute leukemia's an increase in circulating complement is common. As one early study has demonstrated increased expression of complement in monocytes by conditioned media of leukemic cells [48]. At present, a straight forward hypothesis on the functional consequences of the variation in C6 expression in the different forms of HS is not easily postulated but it might be associated with a reaction of the innate immune system to the neoplasm and not a direct effect of the neoplastic cell population.

The variation in expression of *C6*, *CCL5* and *CLEC12A*, all three members of the immune response, is worthy of follow up investigations, with focus upon their character as deranged histiocytes and may include comparisons with the canine reactive histiocytic diseases [1].

When comparing the results of qPCR and microarray the difference in the expression of some genes between STHS and VHS did not attain statistical significance. The variation between the two methods relates to the fact that a microarray experiment is a semi-quantitative screening method and the qPCR quantitative. Many methodological factors can lead to a lack of correlation between array results and qPCR measurements [49]. The expression of *CD9* (synonym *MRP-1*; motility related protein 1) in VHS was suppressed as compared to STHS at a level trending towards significance (*P* = 0.07) and further investigation of this gene in these sarcomas seems warranted in view of associations with reduced expression of this gene with tumor aggressiveness such as reported for other cancers. A lower expression of *CD9* was found to be associated with the formation of bony metastases in studies using breast cancer cell lines [50], [51]. Similarly, in human colon cancer patients, patients that lacked *CD9* mRNA expression, had a worse prognosis than the cases that did express *CD9* mRNA [52].

DAVID pathway analyses of the significantly differentially expressed genes in the micro array did not lead to the detection of altered expression of whole pathways. Also the genes chosen for qPCR confirmation did not appear to be related at the level of regulation.

The alterations in gene function as detected in the current analyses, need follow up by subsequent investigations by use of antibodies - most still need to be developed and validated for use in the dog to examine an altered expression at protein level.

When looking at tumor conditions in the human that share features with canine HS, none of the eleven genes for which altered function was observed in the present study, have been recognized as aberrantly expressed in micro-array/qPCR studies in Langerhans cell histiocytosis [53].

## Conclusions

As a valuable addition to our previous study, in which we were able to provide evidence for involvement of several genes in the development of HS, irrespective of form, this study provides the most comprehensive database to date of genetic variations in the two most common forms of HS, namely VHS and STHS. Using fold-change analysis, it reveals genetic variations not previously associated with these two forms.

On the basis of quantitative differences in expression of *C6* (up-regulated in VHS versus STHS), *CLEC12A* and *CCL5* (down-regulated in VHS versus STHS) were associated with each subtype. Down-regulation of *CLEC12A* in VHS, the more aggressive form of HS, is in line with previously published observations that such reduced function facilitates migratory capacity of myeloid cells in humans [46]. Down-regulation of *CCL5* is in line with several studies in human cancers [37], [47], however contradictory to others [43]. Further investigations should focus on changes of gene function at protein level and a comparison of these histiocytic malignancies in dog and human.

## Supporting Information

Table S1 **All 191 probes significantly differentially expressed.** Of four of the 191 probes, the annotation could not be traced back. Of the remaining probes, 28 have a ‘genomic’ location for which no gene could be mapped at this moment. 159 Probes have a gene-name, of which 142 are unique. From the selection of most significant (*P*<0.003), unique probes with a threshold of log2 fold change of 1.5 (n = 19), eight were excluded; either for comprising chromosomal regions (n = 3) or clones that did not align (n = 5, marked with *). The eleven genes that remained, are written in bold and italic.
(DOCX)
