# Supplementary material for: The Two Main Forms of Histiocytic Sarcoma in the Predisposed Flatcoated Retriever Dog Display Variation in Gene Expression
Source: PLoS One. 2014 Jun 2;9(6):e98258. doi: 10.1371/journal.pone.0098258 (PMC4041757; doi:10.1371/journal.pone.0098258)
Supplement: Table S1 — All 191 probes significantly differentially expressed. Of four of the 191 probes, the annotation could not be traced back. Of the remaining probes, 28 have a ‘genomic’ location for which no gene could be mapped at this moment. 159 Probes have a gene-name, of which 142 are unique. From the selection of most significant (P<0.003), unique probes with a threshold of log2 fold change of 1.5 (n = 19), eight were excluded; either for comprising chromosomal regions (n = 3) or clones that did not align (n = 5, marked with *). The eleven genes that remained, are written in bold and italic. (DOCX) [file pone.0098258.s001.docx]

| **ENSID** | **geneSymbol** | **M-Fold** | ***P*-value** |
| --- | --- | --- | --- |
| ENSCAFG00000002314 | SCIN * | -3.0945074 | 0 |
| ENSCAFG00000032005 |  | -2.8290905 | 0 |
| ENSCAFG00000017138 | CILP * | -2.7669955 | 0 |
| ENSCAFG00000031355 |  | -2.393319 | 0.016 |
| ***ENSCAFG00000025113*** | ***CLEC12A*** | ***-1.8322875*** | ***0*** |
| ***ENSCAFG00000018171*** | ***CCL5_CANFA*** | ***-1.6869771*** | ***0*** |
| ***ENSCAFG00000018163*** | ***CAMK2A*** | ***-1.6740242*** | ***0*** |
| ***ENSCAFG00000002307*** | ***ASPN*** | ***-1.6688222*** | ***0.0006*** |
| ***ENSCAFG00000015172*** | ***CD9*** | ***-1.5537057*** | ***0.0006*** |
| ***ENSCAFG00000019451*** | ***TKTL1*** | ***-1.5321822*** | ***0.001*** |
| ENSCAFG00000017227 | GABRG2 * | -1.5228159 | 0.0288 |
| ENSCAFG00000028765 |  | -1.4935809 | 0 |
| ENSCAFG00000010274 | CHI3L1 | -1.4750658 | 0 |
| ENSCAFG00000002440 | ENSCAFG00000002440 | -1.318441 | 0 |
| ENSCAFG00000003663 | GSN | -1.2530028 | 0.001 |
| genomic:9+2536529-2536588 |  | -1.239624 | 0.0002 |
| ENSCAFG00000006953 | PDGFRL | -1.231847 | 0.0044 |
| ENSCAFG00000004617 | APOE_CANFA | -1.2228113 | 0.0006 |
| genomic:17+36515113-36515172 |  | -1.202469 | 0 |
| ENSCAFG00000002440 | ENSCAFG00000002440 | -1.2023628 | 0.0002 |
| genomic:8+21360901-21360960 |  | -1.2006772 | 0 |
| ENSCAFG00000018163 | CAMK2A | -1.1761496 | 0 |
| genomic:19+40967827-40967886 |  | -1.1686643 | 0 |
| ENSCAFG00000015172 | CD9 | -1.1282699 | 0.0002 |
| ENSCAFG00000008755 | SRC | -1.118658 | 0 |
| genomic:21-38554543-38554602 |  | -1.0951082 | 0 |
| ENSCAFG00000017404 | SCPEP1 | -1.0778874 | 0.02 |
| ENSCAFG00000032175 |  | -1.0768189 | 0.001 |
| ENSCAFG00000002440 | ENSCAFG00000002440 | -1.0573947 | 0.0008 |
| ENSCAFG00000014616 | COMP | -1.0537542 | 0.0024 |
| ENSCAFG00000013664 | C15orf43 | -1.0395595 | 0.0196 |
| ENSCAFG00000031417 |  | -1.0255353 | 0 |
| ENSCAFG00000000274 | HEBP2 | -1.0223435 | 0.0004 |
| ENSCAFG00000019451 | TKTL1 | -1.0206829 | 0.0026 |
| ENSCAFG00000031640 |  | -1.0164332 | 0.001 |
| ENSCAFG00000002590 | XM_848293.1 | -1.0157592 | 0.0004 |
| ENSCAFG00000015598 | DHRS7 | -0.98762298 | 0 |
| ENSCAFG00000029265 |  | -0.98631622 | 0 |
| ENSCAFG00000003096 | PM20D2 | -0.97825308 | 0 |
| ENSCAFG00000001290 | ENSCAFG00000001290 | -0.95298099 | 0.0054 |
| ENSCAFG00000001487 | LGALS1 | -0.95214896 | 0 |
| ENSCAFG00000010404 | RNF19B | -0.94824091 | 0.0002 |
| genomic:15-55825856-55825915 |  | -0.9396565 | 0 |
| genomic:7+27176889-27176948 |  | -0.91433605 | 0.0016 |
| ENSCAFG00000004407 | ME3 | -0.91241496 | 0.007 |
| ENSCAFG00000017677 | GGA2 | -0.91195341 | 0.0168 |
| ENSCAFG00000009568 | CLIC6 | -0.91105517 | 0 |
| ENSCAFG00000018510 | LAMP2 | -0.90830964 | 0.0042 |
| ENSCAFG00000017677 | GGA2 | -0.89362488 | 0.0192 |
| genomic:16+16891636-16891695 |  | -0.87292938 | 0 |
| genomic:1+61045224-61045283 |  | -0.870411 | 0 |
| ENSCAFG00000008700 | FNIP2 | -0.86045192 | 0 |
| ENSCAFG00000017556 | TEX14 | -0.8572938 | 0.001 |
| ENSCAFG00000010790 | XM_535675.2 | -0.85307373 | 0.0324 |
| ENSCAFG00000007112 | SDCBP | -0.84282173 | 0.001 |
| ENSCAFG00000006757 | PPFIBP2 | -0.83953928 | 0 |
| ENSCAFG00000006411 | O46601_CANFA | -0.83252132 | 0.001 |
| ENSCAFG00000014678 | Q6J3Q6_CANFA | -0.82056539 | 0.0014 |
| ENSCAFG00000000303 | AIG1 | -0.81778826 | 0.0302 |
| ENSCAFG00000000126 | Q6JDL3_CANFA | -0.79072133 | 0 |
| ENSCAFG00000020166 | ANGPTL2 | -0.79015989 | 0.0242 |
| genomic:20-41738261-41738320 |  | -0.7843711 | 0 |
| genomic:14+27427086-27427127 |  | -0.7836333 | 0.0002 |
| ENSCAFG00000017019 | TMEM219 | -0.77747733 | 0 |
| ENSCAFG00000028636 |  | -0.77539165 | 0.0056 |
|  |  | -0.77428579 | 0.0058 |
| ENSCAFG00000019764 | DENND2D | -0.77328926 | 0.0228 |
| ENSCAFG00000004174 | SPAG6 | -0.76886501 | 0.0236 |
| ENSCAFG00000004606 | BIN1 | -0.75826718 | 0.0008 |
| ENSCAFG00000019552 | HMHA1 | -0.74655767 | 0.001 |
| ENSCAFG00000023898 | MAP7D1 | -0.73637421 | 0 |
| ENSCAFG00000004318 | CST7 | -0.73109856 | 0.023 |
| ENSCAFG00000001445 | GRINA | -0.72694489 | 0.0016 |
| ENSCAFG00000028791 |  | -0.72520758 | 0.0002 |
| genomic:21+39412439-39412498 |  | -0.72404657 | 0.0012 |
| ENSCAFG00000002115 | RELX_CANFA | -0.72124919 | 0.0008 |
| genomic:25+4418859-4418918 |  | -0.70750093 | 0.026 |
| ENSCAFG00000005236 | TRAK1 | -0.70293539 | 0.0068 |
| genomic:14+52078289-52078348 |  | -0.69932801 | 0.001 |
| ENSCAFG00000031320 |  | -0.69881976 | 0.0052 |
| ENSCAFG00000029573 |  | -0.6958488 | 0.0004 |
| ENSCAFG00000008358 | LOXL3 | -0.69577168 | 0.004 |
| genomic:20-37332869-37332928 |  | -0.68757878 | 0 |
| ENSCAFG00000019764 | DENND2D | -0.6827793 | 0.001 |
| genomic:13+23288136-23288195 |  | -0.68183474 | 0.0156 |
| ENSCAFG00000001624 | CYHR1 | -0.68117993 | 0 |
| ENSCAFG00000008268 | MND1 | -0.67872761 | 0.0452 |
| ENSCAFG00000004481 | SC6A6_CANFA | -0.67597748 | 0 |
| ENSCAFG00000003272 | XM_847318.1 | -0.67459747 | 0.0138 |
| ENSCAFG00000031225 |  | -0.67361452 | 0.0184 |
| ENSCAFG00000014643 | GNG2 | -0.67203677 | 0 |
| ENSCAFG00000000309 | FUCA2 | -0.66930852 | 0.001 |
| ENSCAFG00000019694 | GPSM1 | -0.66332814 | 0.001 |
| ENSCAFG00000000309 | FUCA2 | -0.65892967 | 0.0004 |
| genomic:8+42357182-42357241 |  | -0.65570779 | 0.022 |
| ENSCAFG00000019529 | NDUFS7 | -0.65171998 | 0.001 |
| ENSCAFG00000018517 | C17orf79 | -0.64638989 | 0.0006 |
| ENSCAFG00000016583 | MAD2L2 | -0.64505818 | 0.0228 |
| ENSCAFG00000011392 | SSPN | -0.63215035 | 0.0336 |
| ENSCAFG00000032711 |  | -0.63184405 | 0 |
| ENSCAFG00000030152 |  | -0.62921078 | 0.0022 |
| ENSCAFG00000002128 | KIAA1432 | -0.62756937 | 0 |
| ENSCAFG00000012101 | RB22A_CANFA | -0.62317398 | 0.0036 |
| ENSCAFG00000006757 | PPFIBP2 | -0.62189239 | 0.0032 |
| ENSCAFG00000000581 | TFB1M | -0.61478987 | 0.0302 |
|  |  | -0.6140875 | 0.0008 |
| ENSCAFG00000000215 | R3HDM2 | -0.59808779 | 0.0082 |
| ENSCAFG00000004030 | CMPK1 | -0.59773842 | 0.001 |
| ENSCAFG00000011083 | LOC610413 | -0.59746117 | 0.0018 |
| ENSCAFG00000015532 | MYO9B | -0.59053194 | 0.009 |
| ENSCAFG00000005216 | ULK4 | -0.59034078 | 0.0402 |
| genomic:X-18027472-18027517 |  | -0.58322294 | 0 |
| ENSCAFG00000019544 | EDF1 | -0.57857686 | 0.0244 |
| ENSCAFG00000016509 | UBQLN2 | -0.57384476 | 0.0092 |
| ENSCAFG00000032714 |  | -0.57302038 | 0.0192 |
| ENSCAFG00000006757 | PPFIBP2 | -0.5724539 | 0.0018 |
| ENSCAFG00000019476 | NDUFB10 | -0.57187476 | 0.001 |
| ENSCAFG00000018624 | OMG | -0.56684611 | 0.0474 |
| ENSCAFG00000006339 | CACNA2D1 | -0.56672644 | 0.0044 |
| ENSCAFG00000001582 | ENSCAFG00000001582 | -0.56050664 | 0.0326 |
| genomic:6-39440123-39440182 |  | -0.56017477 | 0.001 |
| ENSCAFG00000004652 | BGAL_CANFA | -0.54880524 | 0.001 |
| ENSCAFG00000029941 |  | -0.54738559 | 0.001 |
| ENSCAFG00000031169 |  | -0.54033337 | 0.0222 |
| genomic:5+12102468-12102527 |  | -0.53227813 | 0.006 |
| ENSCAFG00000019476 | NDUFB10 | -0.52966826 | 0.0036 |
|  |  | -0.5204114 | 0.0132 |
| ENSCAFG00000006963 | AAAS | -0.46857512 | 0.0286 |
| ENSCAFG00000017666 | DCTN5 | -0.45944298 | 0.035 |
| ENSCAFG00000012603 | IFT46 | -0.45781328 | 0.0362 |
| ENSCAFG00000000054 | ENSCAFG00000000054 | 0.43544725 | 0.035 |
| ENSCAFG00000010826 | SF3B1 | 0.43701462 | 0.04 |
| ENSCAFG00000016993 | USP3 | 0.46595357 | 0.0254 |
| ENSCAFG00000016784 | DFFA | 0.46718556 | 0.027 |
| ENSCAFG00000001548 | SNAPC3 | 0.4826005 | 0.0238 |
| ENSCAFG00000003471 | PNISR | 0.51075942 | 0.0338 |
| ENSCAFG00000018257 | MARK3 | 0.51729585 | 0.0416 |
| ENSCAFG00000032308 |  | 0.52883133 | 0.0242 |
| ENSCAFG00000013577 | EIF3J | 0.5376539 | 0.008 |
| ENSCAFG00000011434 | UBR1 | 0.56169272 | 0.0322 |
| ENSCAFG00000005847 | ADAM9 | 0.58015513 | 0.0196 |
| ENSCAFG00000016244 | LBR | 0.60587357 | 0.001 |
| ENSCAFG00000002746 | KLHL7 | 0.61073785 | 0.001 |
| ENSCAFG00000017848 | ENSCAFG00000017848 | 0.61218262 | 0.0358 |
| ENSCAFG00000016711 | C5orf25 | 0.6231177 | 0.027 |
| ENSCAFG00000011929 | LOC478866 | 0.63431446 | 0.0242 |
| ENSCAFG00000002860 | SMEK2 | 0.63517859 | 0.001 |
| ENSCAFG00000001331 | ZCCHC6 | 0.63708157 | 0.0234 |
| ENSCAFG00000002860 | SMEK2 | 0.64296953 | 0.0154 |
| genomic:4+43188112-43188175 |  | 0.65168318 | 0.0012 |
| ENSCAFG00000017207 | CCNG1 | 0.65444471 | 0.0036 |
| genomic:26-20200191-20200245 |  | 0.65527855 | 0.0024 |
| ENSCAFG00000001966 | SH3RF3 | 0.68929916 | 0.0252 |
| ENSCAFG00000024406 | XM_846227.1 | 0.70312728 | 0.0008 |
| ENSCAFG00000013158 | XM_545525.2 | 0.77218972 | 0.0256 |
| ENSCAFG00000010826 | SF3B1 | 0.77354067 | 0.001 |
| ENSCAFG00000029757 |  | 0.78390919 | 0.001 |
| genomic:30+2405784-2405842 |  | 0.79089712 | 0.0192 |
| ENSCAFG00000013817 | SRGN | 0.80563916 | 0.0104 |
| ENSCAFG00000014776 | WNK4 | 0.81432893 | 0.0004 |
| ENSCAFG00000009533 | SSRA_CANFA | 0.8224528 | 0.022 |
| ENSCAFG00000005886 | CRIM1 | 0.82833911 | 0.0132 |
|  |  | 0.84532545 | 0.0016 |
| ENSCAFG00000029252 |  | 0.86129638 | 0.001 |
| ENSCAFG00000003710 | AIM1 | 0.87252936 | 0.0024 |
| genomic:4+64463924-64463983 |  | 0.87433271 | 0.003 |
| genomic:9-43727939-43727998 |  | 0.93653807 | 0 |
| ENSCAFG00000013817 | SRGN | 0.94811488 | 0 |
| genomic:X-48093611-48093670 |  | 0.96151221 | 0 |
| ENSCAFG00000011930 | CCNDBP1 | 1.008616 | 0.0002 |
| ENSCAFG00000008999 | PROS1 | 1.0211954 | 0.0016 |
| ENSCAFG00000013310 | ENSCAFG00000013310 | 1.0406891 | 0 |
| ENSCAFG00000008341 | ENSCAFG00000008341 | 1.0465609 | 0 |
| ENSCAFG00000010595 | RGS2 | 1.0808145 | 0.0068 |
| ENSCAFG00000000460 | LAPTM4B | 1.091016 | 0.0486 |
| ENSCAFG00000010595 | RGS2 | 1.1236993 | 0.009 |
| ENSCAFG00000006371 | NRG1 | 1.1358697 | 0.001 |
| ENSCAFG00000018258 | ENSCAFG00000018258 | 1.2283262 | 0 |
| ENSCAFG00000008205 | ENSCAFG00000008205 | 1.2448566 | 0.0224 |
| ENSCAFG00000010877 | ABCA9 | 1.2480915 | 0.0164 |
| ENSCAFG00000024916 | ABCA8 | 1.4009914 | 0.0014 |
| ENSCAFG00000029470 |  | 1.4653424 | 0.0154 |
| ENSCAFG00000018598 | C6 | 1.5415842 | 0 |
| ENSCAFG00000008291 | Q9TU80_CANFA * | 1.7023561 | 0 |
| ***ENSCAFG00000023324*** | ***S100A12*** | ***1.7561693*** | ***0.0228*** |
| ***ENSCAFG00000002911*** | ***IGJ*** | ***2.0578904*** | ***0.0052*** |
| ENSCAFG00000023441 | * | 2.0670019 | 0.001 |
| ***ENSCAFG00000017557*** | ***S100A8*** | ***2.2845349*** | ***0.0002*** |
| ENSCAFG00000008291 | Q9TU80_CANFA | 2.3489151 | 0 |
| ***ENSCAFG00000018598*** | ***C6*** | ***2.3790026*** | ***0*** |
| ***ENSCAFG00000023349*** | ***PHYH*** | ***2.9195861*** | ***0.0004*** |
